# A Voluntary National Examining Board

**Published:** 1902-07

**Authors:** 


### A Voluntary National Examining Board.

AT THE recent meeting of the National Confederation of State Medical Examining and Licensing Boards at Saratoga, the question of interstate reciprocity of licensure was again discussed, this time under a new proposition. It has been suggested many times and in several forms that a National Board be created. The impossibility of such action by congress has been pointed out time and again. The reasons are so apparent as to preclude the necessity of further discussion.

Now comes the proposal to establish a voluntary national board made up of seven members—namely, the Surgeon-Generals of the three services, two from the American Medical Association, one from the Congress of American Physicians and Surgeons, and one from the National Confederation of State Medical Examining and Licensing Boards. This proposition came before the latter organisation at Saratoga and was referred, after ample discussion, to a committee for a report as to its feasibility. The report is as follows:

In the opinion of your committee this Confederation cannot endorse nor approve such a proposition for the following reasons: (1) A Voluntary National Examining Board would have no power, no authority, nor legal right to exist; (2) no guarantee could be given of the continuance or permanency of such voluntary board, even were the laws of the several states so modified as to meet its requirements; (3) being a voluntary board there could be no legal manner of constituting, changing or limiting its membership, or of defining its duties ; (4) such a board would be representative of the profession only, and of the regular profession alone ; (5) without the endorsement of a state board authorised by law to grant a license to practise, a certificate of qualification from the proposed voluntary board could have no value whatever, and under the existing laws of the several states the state examining boards are required to conduct the examinations, and such boards cannot evade nor surrender such duty, even if they desired to do so ; (6) to attempt the stupendous task of securing the amendments to the existing laws regulating the practice of medicine in the several states would entail enormous labor and expense, and would probably endanger the laws themselves.

*American Medicine*, in its issue of June 14, 1902, makes the following intelligent comment upon the subject:

The Evolutionary Factor in Medical Licensure and Reciprocity.—To those capable of reading between the lines, the adverse report of the National Confederation upon the proposal to establish a Voluntary National Board of Examiners will have a vast significance. We have repeatedly called attention to some of these phases, but all are based upon the fundamental principle of careful evolution by modification and progress along lines indicated by established laws and present facts. There must be evolution, not revolution, in order that finally harm may not be done to all in the effort to do away with temporary and individual hardships and injustice. A voluntary board is simply not feasible and would undoubtedly destroy the substantial progress in reciprocity already attained and the more perfect results that are surely coming by the outworking of present tendencies and aims. It is constantly forgotten that in the long run there can be no absolute settlement of the great problem of reciprocity until there is a consent and practical harmony on the part of all the states as to the standards of preliminary education. This places far in the future the ultimate solution of the question, because the states cannot and, moreover, should not, hurry too much the orderly progress by any revolutionary enactment of remarkable laws. The greatest danger lies in the unit of the Confederation report, that in the present condition of the popular and legislative mind any extensive attempt to tamper with the great body and attainment of medical laws would at once bring the whole structure tumbling to ruins about us. In the meantime, by individual and considerate action, state boards can do a great deal toward extending to incoming older practitioners from other states who are professionally and socially honorable a courteous welcome which shall do away with many instances of injustice which have taken place in the past. These exceptional cases, however, should not make us forget our greater duty to the entire profession and especially to the profession of all the future.

